# The impact of rest breaks on subjective fatigue in physicians of the General Hospital of Vienna

**DOI:** 10.1007/s00508-021-01949-1

**Published:** 2021-10-06

**Authors:** Gerhard Blasche, Anna Arlinghaus, Richard Crevenna

**Affiliations:** 1 Center for Public Health, Department of Environmental Health, Medical University of Vienna, Kinderspitalgasse 15, 1090 Vienna, Austria; 2 XIMES GmbH, Vienna, Austria; 3 Department of Physical Medicine, Rehabilitation and Occupational Medicine, Medical University of Vienna/General Hospital of Vienna, Vienna, Austria

**Keywords:** Subjective fatigue, Work Breaks, Burnout, Hospital physicians, Day shift

## Abstract

**Aims:**

Medical doctors are affected by high levels of occupational burnout. Work organization such as sufficient rest breaks can decrease stress and fatigue; however, rest breaks are often skipped under high workload and time pressure. The present study sought to investigate the effect of self-determined rest breaks on acute and daily levels of fatigue in physicians of the General Hospital of Vienna.

**Methods:**

Rest breaks and fatigue were recorded throughout day shifts over a time span of 4 weeks with a mobile assessment device. A total of 12 physicians of a university clinic participated in the study. Data were analyzed using generalized estimating equations.

**Results:**

Analyses were based on a total of 115 workdays including 93 rest breaks and 800 fatigue assessments. Physicians took an average of 0.81 rest breaks per day. Fatigue was lower in the 30 min after the break than in the hour before the break; however, the number of rest breaks did not affect the increase of fatigue during shifts.

**Conclusion:**

Self-determined rest breaks were effective in reducing acute fatigue in hospital physicians during work. The failure to find an effect on the increase of work-related fatigue may be due to the infrequency of rest breaks in hospital physicians.

## Introduction

Physicians are faced with high levels of burnout, a syndrome primarily based on emotional exhaustion (i.e. mental and motivational fatigue) and depersonalization (i.e. a negative attitude towards patients and work) [1]. Studies have found that the prevalence of burnout in physicians significantly exceeds that of the general public [2] and has increased in the last decade in various countries [3, 4]. High levels of burnout also have been found in Austrian physicians [5], levels being comparable, albeit slightly lower, to those found in an international meta-analysis [6]. Consequences of physician burnout are severe, including an increased risk of patient safety, poorer quality of care, reduced patient satisfaction, lower productivity and lower levels of physician health [1, 7–9]. Several factors have been identified to contribute to burnout in physicians, including inefficient work processes, excessive workload, bad organizational climate and family responsibilities [1, 10].

Based on this situation recently described as “global crisis of physician burnout” by the Lancet [11], efforts to reduce burnout in physicians have been undertaken. Interventions aimed at the physician (e.g. stress management) and interventions aimed at the organization (e.g. focusing workload, work schedule or communication) are effective in reducing burnout, although those addressing the organization alleviate burnout to a greater degree [12, 13]. One measure potentially reducing burnout are rest breaks. Rest breaks can be seen as determined both by organizational as well as by individual factors [14, 15]. It can be assumed that rest breaks prevent the build-up of acute fatigue and thus reduce the risk of exhaustion on the long run, despite the fact that fatigue and burnout are overlapping, but not identical conditions [16, 17]. Rest breaks have been shown to reduce subjective fatigue [18, 19], improve performance and work engagement [20, 21] and reduce occupational injuries and physical complaints [22, 23]. For nurses, rest breaks are acknowledged to be important to sustain health and well-being [14]; however, despite some studies on the positive effect of rest breaks on surgeons during surgery [23, 24], rest breaks have not been studied in medical doctors to the best of our knowledge, which is surprising considering the elevated levels of burnout in this group of health professionals.

In the present study, the effect of rest breaks on fatigue was investigated in physicians of a University Clinic of the General Hospital of Vienna during day shifts. The present study utilized a design with high ecological validity, assessing self-determined rest breaks and fatigue throughout the workday over a time span of 4 weeks. For this purpose, a mobile device with minimal impact on working routine was used. We assumed that (hypothesis 1) subjective fatigue would be lower after a rest break than prior to a rest break, (hypothesis 2) subjective fatigue would increase over the workday, and (hypothesis 3) that the increase of fatigue over a working day would be negatively related with the number of rest breaks taken during that day.

## Material and methods

### Study participants

Study participants were 12 physicians (5 females, 7 males, mean age = 38.6 years, SD 7.2 years) of the Department of Physical Medicine, Rehabilitation and Occupational Medicine of the General Hospital of Vienna working more than 35 h per week. Study participants only worked day shifts. Study participants were recruited by contacting all 17 department physicians via email and inviting them to participate in a study on the effect of rest breaks on fatigue. Information regarding the study was provided during a meeting. All study participants provided written informed consent.

### Design and procedure

Study participants received a mobile device (XIMES corp. Vienna, Austria) [25] for recording rest breaks as well as fatigue during workdays for a time span of 4 weeks. Individuals recorded the start and end of individually paced rest breaks defined as temporary interruptions of work for the means of recovery with a duration of at least 3 min. No restrictions were placed on the number, time and duration of rest breaks, which were fully at the individual’s discretion. In addition, individuals were prompted roughly every 90 min by a vibration of the device to record their present level of fatigue with a single item measure. Fatigue was also assessed at the beginning and end of each workday. Every fatigue assessment was recorded with a time stamp. The beginning and end of workdays were determined by activating and deactivating the device by the study participant. The assessment took place from 5 to 27 November 2019. The study was approved by the ethics board of the Medical University of Vienna (EK Nr. 2020/2018).

### Variables

The dependent variable was acute fatigue assessed with the single item measure “How much in need of recovery do you feel at the present moment?”. This item was chosen from the items of the fatigue sub-scale from a standardized German well-being questionnaire [26] on the basis of previous diary research [27, 28] as it showed the highest levels of change sensitivity. Answers were coded on a worded numeric rating scale with the descriptors 1 = “not at all” (“gar nicht”), 2 = “hardly” (“kaum”), 3 = “slightly” (“leicht”), 4 = “averagely” (“mittel”), 5 = “fairly” (“ziemlich”), 6 = “very” (“sehr”) and 7 = “extraordinarily” (“außerordentlich”). The fatigue measure showed an acceptable distribution (m = 3.9, SD = 1.8, skewness = 0.08, kurtosis = −0.79, range = 1–7). The independent variables were the number of rest breaks per day (rest break frequency), the time of day as well as the relative time between rest breaks and fatigue assessments. Additional variables were the number of assessment days and workdays per individual, the beginning and end of workday and the duration of workdays.

### Data analysis

The original dataset included a total of 172 observation days with a total of 986 fatigue assessments and 118 rest breaks. Of these, 5 days were excluded because they did not contain any fatigue assessments. An additional 39 days with less than 5 daily fatigue assessments were excluded to provide sufficient data for estimating daily means and changes of fatigue. In addition, 13 short workdays, i.e. workdays starting after 9:00 h and/or ending before 14:00 h, were excluded, leaving a total of 115 days for the final analyses, including a total of 800 fatigue assessments. Of the rest breaks 8 were excluded because of durations shorter than 3 min, leaving a total of 93 rest breaks. In a first step, secondary variables such as the number and duration of workdays per person, the number of rest breaks per day and the duration of rest breaks were determined from the primary variables using Excel formulas. Data were then analyzed with SPSS 26 (IBM Corp.) using the procedure generalized estimating equations. Calculations were based on a linear model using a robust estimator adjusted by the number of nonredundant parameters and an independent working correlation matrix. A subject variable was entered in every model. To evaluate the acute effect of rest breaks, levels of fatigue 0–30, 31–60 and 61–90 min following the rest break were compared to levels of fatigue in the hour preceding the rest break (entered as categorical variable). To control for time of day effects, the hour of day was added as covariate (entered as continuous variable). To evaluate the effect of the number of rest breaks per day on the level and change of fatigue on a given day, fatigue was predicted by a modified rest break frequency variable as described below as well as the time of day and an interactive term to evaluate the change of fatigue as a consequence of rest breaks (entered as categorical variables). Effects sizes (Cohens d) were calculated with the estimated means and the standard errors transformed to standard deviations.

The original rest break frequency variable (i.e. number of rest breaks per day) was unsuitable for analyses as some categories contained 5 or fewer entries, which can be considered insufficient for analysis. In addition, rest breaks were quite unequally distributed between study participants, the average number of rest breaks per day varying between 0 and 3 rest breaks per participant and the total number of days with rest breaks varying between 0% (2 individuals) and 100% (4 individuals). Therefore, a new rest break variable (rest break variation) was determined to be able to observe the intra-individual effect of the variation of rest breaks encompassing two categories. This variable compared the minimum number of rest breaks per day (which was 0 for 8 individuals, 1 for 3 individuals and 2 for 1 individual, coded as “0”) with all other days with a larger number of rest breaks (coded as “1”). In effect, the variable rest break variation compared days with fewer or no rest breaks (j, where 0 ≤ j ≤ 2, coded as 0) with days with one or more additional rest breaks (j + k, where 1 ≤ k ≤ 3, coded as 1).

## Results

The average number of observation days (i.e. workdays) per person was 9.6 days (SD 4.5 days, range 2–16), the number of fatigue assessments was 66.7 assessments (SD 33.3 assessments, range 15–120). The average duration of workdays was 8.2 h (SD 0.91h). Individuals started their workday at 7:46 (SD 0:31) and ended it at 15:55 (SD = 0:47). An average of 0.81 rest breaks (SD 1.0) per day was observed, with a minimum of 0 and a maximum of 5 breaks. The majority of breaks (41%) were taken at lunchtime (12:00–13:59 h), 22% in the mid-morning between 10:00–11:59 h, 14% in the early morning between 7:00–8:59 h and 23% in the afternoon after 13:00 h. The average duration of rest breaks was 23.0 min (SD 14.1 min), varying between 3 and 48 min, 46 breaks (49%) were longer than 20 min and thus can be considered as lunch breaks.

**Fig. 1:**
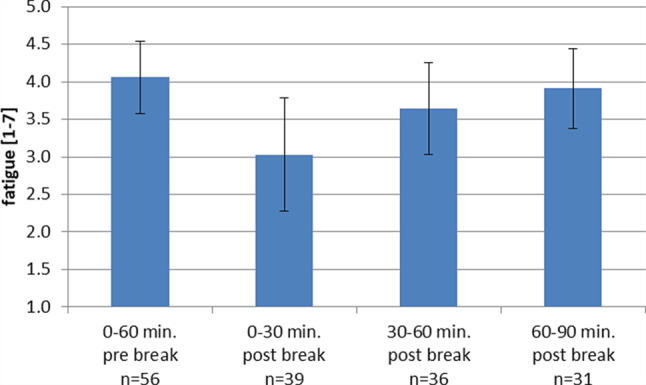
Acute effect of rest breaks on subjective fatigue; estimated means (corrected for hour of day) and 95% confidence intervals are displayed; *n* is the number of observations

The acute effect of self-determined rest breaks is illustrated in Fig. 1. Fatigue was lower in the 30 min after the break than in the hour before the break (B = −1.03, SE = 0.21, *p* < 0.001), with a medium effect size of d = 0.49. A near significant difference to the pre-break level was found for fatigue 31–60 min after the break (B = −0.42, SE = 0.23, *p* =0.076; d = 0.23), whereas 60–90min after the break levels of fatigue did not differ from the pre-break level (B = −0.15, SE = 0.33, *p* =0.65). This result supports hypothesis 1. The hour of day as control variable was positively related to fatigue (B = 0.23, SE = 0.10, *p* = 0.017). The goodness of fit of this analysis was QIC (Quasi Likelihood under Independence Model Criterion) = 327.

**Fig. 2:**
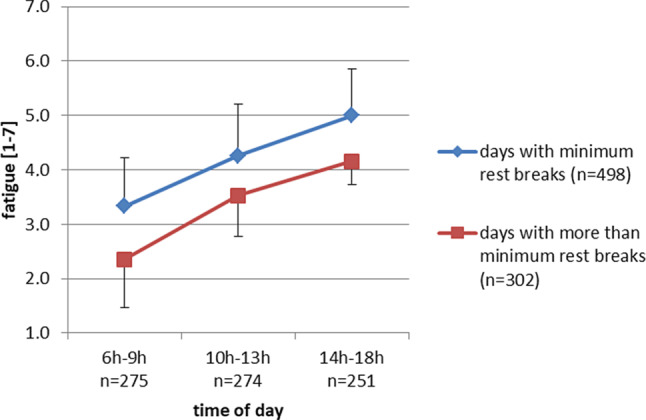
Effect of rest break variation on subjective fatigue during workdays; estimated means (corrected for hour of day) and 95% confidence intervals are displayed; *n* is the number of observations

The effect of the time of day and rest breaks on fatigue during workdays is illustrated in Fig. 2. Fatigue increased significantly during a workday (*p* < 0.001; B(midday: 10–13 h) = 0.94, SE = 0.18, *p* < 0.001; B(afternoon: 14–18 h) = 1.67, SE = 0.25, *p* < 0.001; reference cathegory: morning (6–9 h)), thus supporting hypothesis 2. The overall effect of rest break variation (i.e. days with the minimum number of rest breaks per person versus days with more than the minimum number of rest breaks) on overall fatigue during workdays was significant (*p* = 0.033; B(additional breaks) = −0.98, SE = 0.51; reference cathegory: minimum rest breaks). Fatigue generally was lower on days where individuals took more breaks; however, rest break variation did not affect the *change* of fatigue during a workday (*p* =0.27; B(additional breaks × midday) = 0.24, SE = 0.19; B(additional breaks × afternoon) = 0.13, SE = 0.35; reference cathegory: additional breaks × morning). Thus, hypothesis 3 was not supported. The goodness of fit of this analysis was QIC = 2121.

## Discussion

Medical doctors are faced with high levels of burnout, a syndrome characterized among others by exhaustion. A measure known to alleviate fatigue and thus potentially preventing burnout are rest breaks, the temporary interruptions of work for the means of recovery. The present study sought to investigate the effect of self-determined rest breaks on subject fatigue in physicians working at a university clinic.

Self-chosen rest breaks were associated with an immediate decrease in fatigue in the 30 min following the break as compared to the 60min before the break, indicating that rest breaks were effective in reducing fatigue during routine work. Fatigue increased in the next 30min following the break, with a level still marginally below pre-break levels. An hour after the break fatigue was non-significantly different from pre-break levels. These effects are comparable to those of other studies showing that rest breaks acutely reduce fatigue in natural work settings [18, 19]; however, the present study is the first to suggest that the effect of rest breaks on subjective fatigue dissipates in the 90 min following the break, when work is resumed.

In the present study, subjective fatigue increased over the workday, indicating that work was indeed tiring. An increase in fatigue during real or simulated work in various occupations also has been found in several other studies [29–31]; however, in contrast to these studies, where fatigue increased predominantly in the afternoon, morning and afternoon increases in fatigue were of similar magnitude in the present study, possibly reflecting high morning levels of workload. An alternative explanation for the observed morning increase in fatigue is that individuals showed low levels of burnout and/or high sleep quality [29, 31]; however, this latter explanation is unlikely keeping in mind the high levels of burnout reported for physicians [2].

Contrary to expectation, rest breaks did not have an effect on the change of fatigue during the workday. The time course of fatigue was similar on days individuals took one or more rest breaks compared to days when individuals took fewer or no rest breaks. This result differs from the results of a study on driving examiners showing that taking rest breaks at regular intervals during the workday was associated with lower levels of post-work fatigue compared to not taking any rest breaks [32]. Similarly, it is in contrast to a study showing that nurses who were more inclined to take rest breaks had lower levels of fatigue at the end of their work shift [15]. A reason for the failure to find positive results on the change of fatigue may be the generally low number of breaks observed in the present study. In contrast to the average of 0.9 breaks per day observed here, other studies report an average number of rest breaks between 2.5 [18] and 2.9 [15] per day. Despite the large differences in the taking of rest breaks between individuals in the present study, even the number of rest breaks in those taking more breaks may have been insufficient to affect the time course of fatigue. Another reason may lie in the timing of rest breaks. It has been shown that breaks taken in the afternoon have a greater impact on fatigue than those taken in the morning [21]. In the present study, however, only few breaks were taken in the afternoon, thus potentially limiting their effect on fatigue.

Despite the failure to find an effect of breaks on the change of fatigue over the workday, there was an effect on the mean levels of daily fatigue. Individuals taking more rest breaks had lower levels of fatigue during the workday. This is in line with the results of a study on hospital physicians showing that the adherence to designated breaks was associated with lower levels of emotional exhaustion [33]; however, as fatigue tends to accumulate over a workday as indicated, one would expect rest breaks to have a greater impact on fatigue when fatigue levels are higher. Possibly, the effect of rest breaks on mean levels of fatigue is a result of interindividual differences in study participants, for example regarding gender [34] or work engagement [35]. Future studies will have to assess these individual differences to be able to clarify this issue.

The participants of the present study took considerably fewer rest breaks than observed in studies conducted with predominantly administrative employees [15, 18]. These differences cannot be accounted for by different assessment methods, as all studies assessed breaks using diaries or experience sampling. Thus, it is reasonable to assume that they are associated with professional and/or occupational characteristics. For example, the majority of surgeons appraise intraoperative breaks negatively, despite their documented benefits for both the surgeon and the patient [36]. Although taking breaks during a surgical procedure is a special case, it may also reflect doctors’ attitude towards work breaks in general, suggesting that physicians are more reluctant to take breaks than members of other professions. In addition, occupational characteristics in healthcare may impede the taking of breaks due to higher levels of occupational demands and lower levels of control [37].

It is worth noting that the number of days individuals recorded data differed strongly between individuals. Although these differences in study participation may be due to factors not associated with the study (sick leave, vacation, other work constraints), they may also reflect differences in study adherence; however, due to the small number of study participants it was not possible to evaluate if these potential differences in study adherence affected outcomes in the present study by for example the omission to record rest breaks.

The strength of the present study is the use of a mobile assessment system, which allowed us to track rest breaks as well as subjective fatigue during a workday with objectively assessed clock times. Limitations of the present study are the relatively small number of study participants and observed rest breaks, reducing overall variance and limiting the power of the study. To achieve a greater statistical power and to enable a more finely tuned analysis, for example evaluating the effect of a broader range of daily rest breaks or differences between morning and afternoon breaks, the study would need to be replicated with a larger number of study participants. A second related limitation are the large interindividual differences in observation days, leading to an unequal contribution of study participants to the overall results.

To conclude, rest breaks acutely reduce fatigue in hospital physicians during day shifts. Taking more rest breaks was also associated with lower levels of overall fatigue during dayshifts; however, rest breaks did not dampen the increase of fatigue during shifts. Possibly, the failure to counteract increases in fatigue is due to the infrequency of breaks observed in medical doctors. Thus, although the results suggest that rest breaks reduce fatigue on a short-term basis, future studies with larger sample sizes will have to ascertain whether rest breaks can counteract the increase in fatigue during a work shift and thus provide a means for preventing professional burnout in physicians.
